# Circulating MicroRNA-122 for the Diagnosis of Hepatocellular Carcinoma: A Meta-Analysis

**DOI:** 10.1155/2020/5353695

**Published:** 2020-03-23

**Authors:** Xiao-Fei Zhao, Ning Li, Dong-Dong Lin, Li-Bo Sun

**Affiliations:** ^1^Department of General Surgery and Liver Transplantation Center, Capital Medical University Affiliated Youan Hospital, Beijing 100069, China; ^2^Capital Medical University Affiliated Youan Hospital, Beijing 100069, China

## Abstract

**Background:**

Circulating microRNA-122 (miR-122) has been recognized as a marker of hepatocellular carcinoma (HCC). The current meta-analysis was performed to quantitatively evaluate the diagnostic performance of circulating miR-122 for HCC.

**Methods:**

Related studies that evaluated the diagnostic performance of circulating miR-122 determined from pathophysiological examination for HCC were obtained by systematic searches of the PubMed and Embase databases. A randomized fixed effects model was applied according to the heterogeneity among studies. The pooled sensitivity, specificity, and area under the summary receiver operating characteristic curve (AUC) were calculated to evaluate the diagnostic accuracy. Publication bias was detected by Deeks' funnel plot asymmetry test.

**Results:**

Thirteen studies providing data for 920 HCC patients and 1217 controls were included in the meta-analysis. The pooled sensitivities, specificities, and AUCs of serum miR-122 were 0.76, 0.75, and 0.82, respectively, for discriminating HCC patients from overall controls; 0.85, 0.83, and 0.91, respectively, for discriminating HCC patients from healthy controls; 0.79, 0.82, and 0.87, respectively, for discriminating HCC from HBV or HCV infection; and 0.65, 0.75, and 0.74, respectively, for discriminating HCC from liver cirrhosis or dysplastic nodule formation. No significant publication bias was detected.

**Conclusions:**

Serum miR-122 confers moderate efficacy for discriminating HCC patients from healthy controls or patients with HBV or HCV infection, but not for discriminating HCC patients from those with liver cirrhosis or dysplastic nodule formation.

## 1. Introduction

Hepatocellular carcinoma (HCC) accounts for more than 80% of cases of primary liver cancer [1], and the prognosis of patients with HCC depends on the staging of the disease. The 5-year survival of patients with early-stage HCC was reported to be as high as 75%, whereas the 1-year survival of patients with widespread HCC was reported to be less than 10%, which highlights the importance of early diagnosis [1, 2].

Currently, clinical screening strategies for HCC mainly involve ultrasonography [3, 4]. However, the diagnostic performance of ultrasonography for early HCC is poor due to its low sensitivity [5]. Other imaging modalities such as computer tomography (CT) and magnetic resonance imaging (MRI) have limited value for HCC screening because of the risk of radiation exposure or high expense [3, 4]. Serum biomarkers may be convenient and effective for early diagnosis of HCC. However, increased circulating alpha fetoprotein (AFP), the most commonly used circulating biomarker for HCC, has been observed in patients without HCC, such as those with chronic hepatitis B or C, suggesting the poor specificity of AFP for HCC [3, 6].

Recent advances in biomedical research have demonstrated that microRNAs (miRNAs), an endogenous group of small noncoding RNAs that negatively regulate gene expression at the posttranscriptional level, are involved in many cellular processes, including carcinogenesis [7]. miRNAs are highly stable and measurable from various tissues, including the peripheral circulation, which makes them potential biomarkers for cancer screening in clinics [8, 9]. MicroRNA-122 (miR-122) has been demonstrated to be involved in the process of hepatocarcinogenesis, and differences in the circulating level of miR-122 have been observed in HCC patients compared with control individuals [8, 10]. Although accumulating studies have evaluated the potential diagnostic performance of miR-122 for HCC, quantitative meta-analyses that summarize the diagnostic efficacy of miR-122 for HCC are still needed. In a previous meta-analysis that included four studies [11–14], Huang et al. concluded that serum miR-122 confers promising diagnostic efficacy for HCC [15]. However, due to the small number of studies included, they were unable to evaluate the efficacy of circulating miR-122 for discriminating HCC patients from various control patient populations [15]. Since the publication of their meta-analysis, several additional studies regarding the diagnostic efficacy of miR-122 for HCC have been published [16–24]. Therefore, we aimed to quantitatively evaluate the potential diagnostic performance of circulating miR-122 for HCC in an updated meta-analysis. Moreover, we explored whether circulating miR-122 confers similar efficacy for discriminating HCC patients from healthy controls, HCC patients from those with HBV or HCV infection, and HCC patients from those with liver cirrhosis.

## 2. Methods

### 2.1. Database Searches

This systematic review and meta-analysis was performed following the Preferred Reporting Items for Systematic Reviews and Meta-Analyses (PRISMA) statement [25] and Cochrane Handbook [26]. The literature reports were obtained via electronic searches of the PubMed (MEDLINE) and Embase databases using the following terms: “microRNA-122”, “miRNA-122”, or “miR-122”, combined with “hepatocellular cancer”, “hepatocellular tumor”, “hepatocellular carcinoma”, “hepatocellular neoplasm”, “liver cancer”, “liver tumor”, “liver carcinoma”, “liver neoplasm”, or “HCC”, with a limitation of studies in humans. The date of the final database search was March 12, 2019. The reference lists of relevant original and review articles were manually searched for potential studies.

### 2.2. Inclusion and Exclusion Criteria

The following inclusion criteria were applied: (1) full-length article published in English; (2) histopathological examination used as the referenced standard for HCC diagnosis; (3) studies aimed at evaluating the diagnostic performance of circulating miR-122 for HCC diagnosis; and (4) reported data being adequate for extraction or calculation of the true- and false-positive values as well as true- and false-negative values for HCC diagnosis with miR-122 as determined by histopathological examination. Review articles, repeated reports, and preclinical studies were excluded.

### 2.3. Data Extraction and Quality Assessment

The literature search, data extraction, and quality assessment were independently performed by two authors, with discrepancies resolved by discussion with the corresponding author. The following data were extracted: name of the first author; year of publication; study location; number, age, and gender of HCC patients and controls; characteristics of controls; and miR-122 sampling and measurement methods. True- and false-positive data and true- and false-negative data for the diagnosis of HCC based on miR-122 were extracted or calculated for meta-analysis. The quality evaluation was performed with the QUADAS (quality assessment tool for diagnostic accuracy studies) scale [27]. The QUADAS scale is a validated tool for quality evaluation of the diagnostic accuracy studies, with the highest score of 14 indicating optimal study quality.

### 2.4. Statistical Analysis

The summary sensitivity and specificity were calculated from 2 × 2 forms with corresponding 95% confidence intervals (CIs). The area under the receiver operating characteristic (AUC) curve derived from the data was taken to reflect the overall effectiveness of each quantitative method. Interstudy heterogeneity was formally tested using Cochran's *Q* test, and significant heterogeneity was defined as *P* < 0.10. We also examined the *I*^2^ statistic to reflect the heterogeneity of the included studies, which describes the percentage of total variation across studies that is due to heterogeneity rather than chance. An *I*^2^ > 50% was considered indicative of significant heterogeneity [28]. In cases of significant heterogeneity as reflected by *I*^2^ > 50%, a random effects model was used to estimate the overall effect; otherwise, a fixed effects model was applied. Because the characteristics of controls may affect the diagnostic efficacy of circulating miR-122 for HCC, we subsequently analyzed the performance of miR-122 for discriminating HCC patients from healthy controls, from patients with HBV or HCV infection, and from patients with liver cirrhosis or dysplastic nodule formation. Deeks' funnel plot asymmetry test was used to evaluate publication bias. Statistical analyses were performed using Stata 12.0. All statistical tests were two-sided, with *P* < 0.05 indicating statistical significance.

## 3. Results

### 3.1. Studies Identified by Database Searches

**Figure 1:**
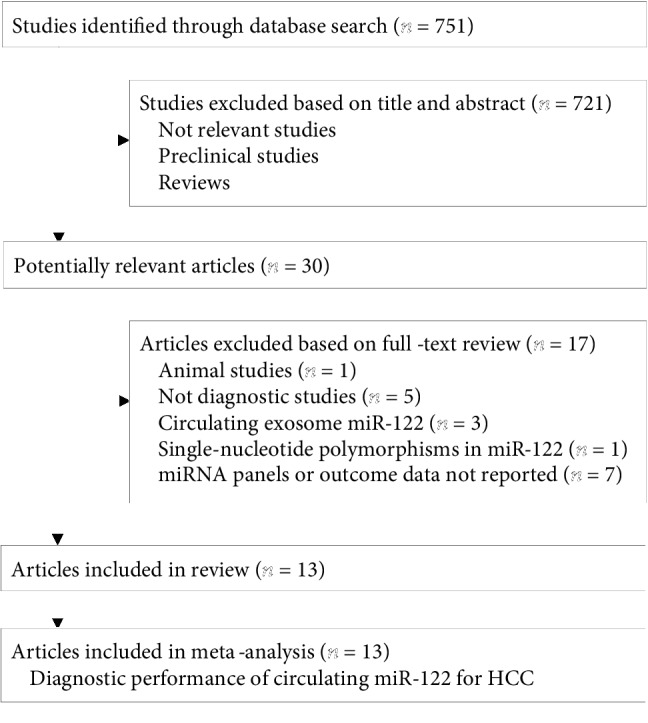
Summary of database searching and study identification.

The processes of database searching and study identification are summarized in Figure 1. Briefly, 751 studies were obtained from database searches, and 30 studies were kept after exclusion of 721 studies based on title and abstract screening for relevance. Seventeen studies were further excluded after full-text review, because they were animal studies (*n* = 1), not designed as diagnostic studies (*n* = 5), reported exosomal miR-122 expression rather than circulating miR-122 expression (*n* = 3), evaluated single nucleotide polymorphisms of miR-122 (*n* = 1), or reported diagnostic efficacies with no available data for miR-122 (*n* = 7). Finally, 13 studies were included [11–14, 16–24].

### 3.2. Study Characteristics and Quality Evaluation

The characteristics of the included studies are summarized in Table 1. Overall, 13 studies reporting data for 920 patients with histopathologically confirmed HCC and 1217 control individuals were included. Seven of the studies were performed in China [11–14, 17, 21, 23], three in Egypt [16, 18, 19], one in Vietnam [22], one in Italy [20], and one in Australia [24]. The characteristics of the control populations were mixed, with inclusion of healthy controls, participants with HBV or HCV infection, and patients with liver cirrhosis or dysplastic nodules. All of the included studies measured serum miR-122 expression by quantitative real-time PCR. The QUADAS scale scores were between 6 and 12 for the included studies, indicating moderate study quality.

### 3.3. Meta-Analysis of the Value of Circulating miR-122 for HCC Diagnosis

**Figure 2:**
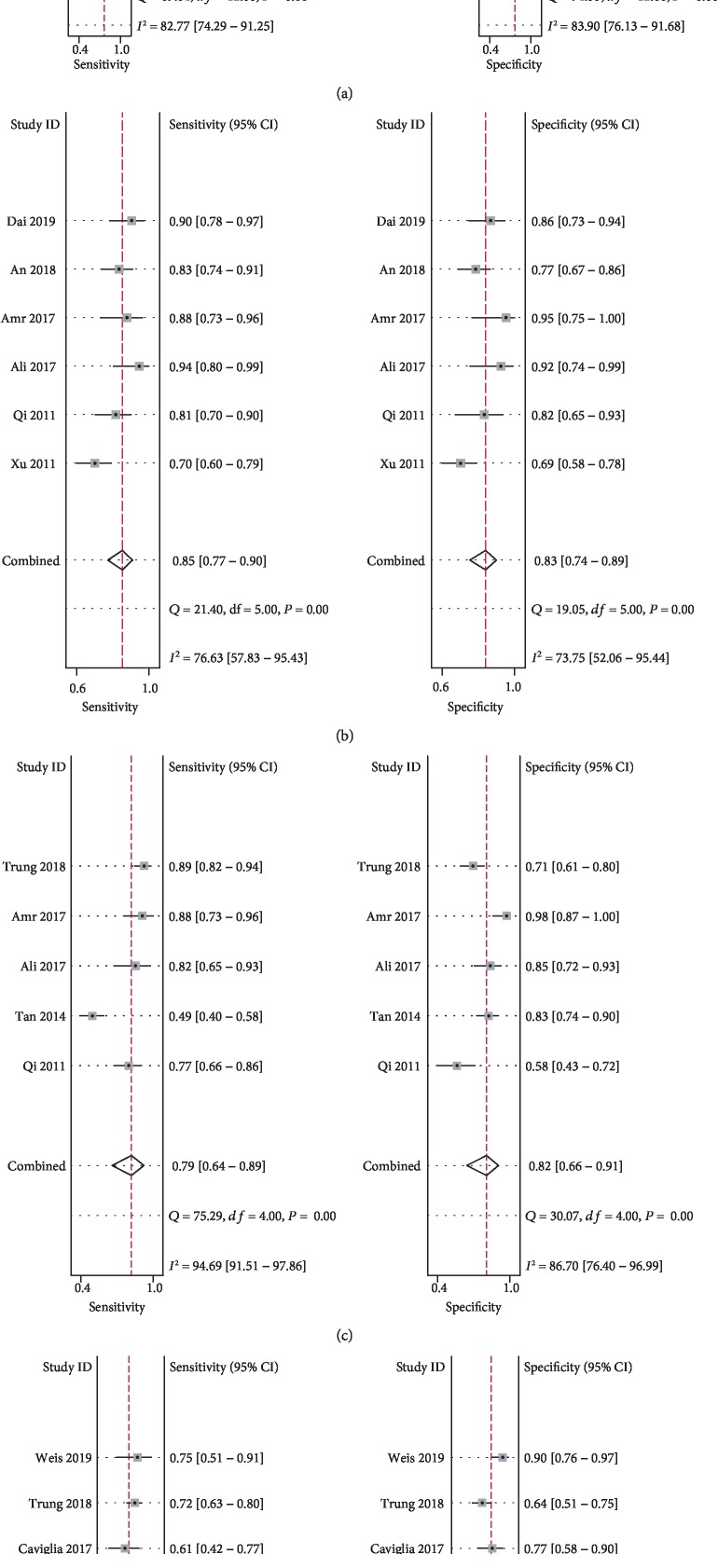
Forest plots for the diagnostic performance of circulating miR-122 for HCC: (a) summarized sensitivity and specificity of serum miR-122 for discriminating HCC patients from overall controls; (b) summarized sensitivity and specificity of serum miR-122 for discriminating HCC patients from healthy controls; (c) summarized sensitivity and specificity of serum miR-122 for discriminating HCC patients from patients with HBV or HCV infection; (d) summarized sensitivity and specificity of serum miR-122 for discriminating HCC patients from patients with liver cirrhosis or dysplastic nodule formation.

**Figure 3:**
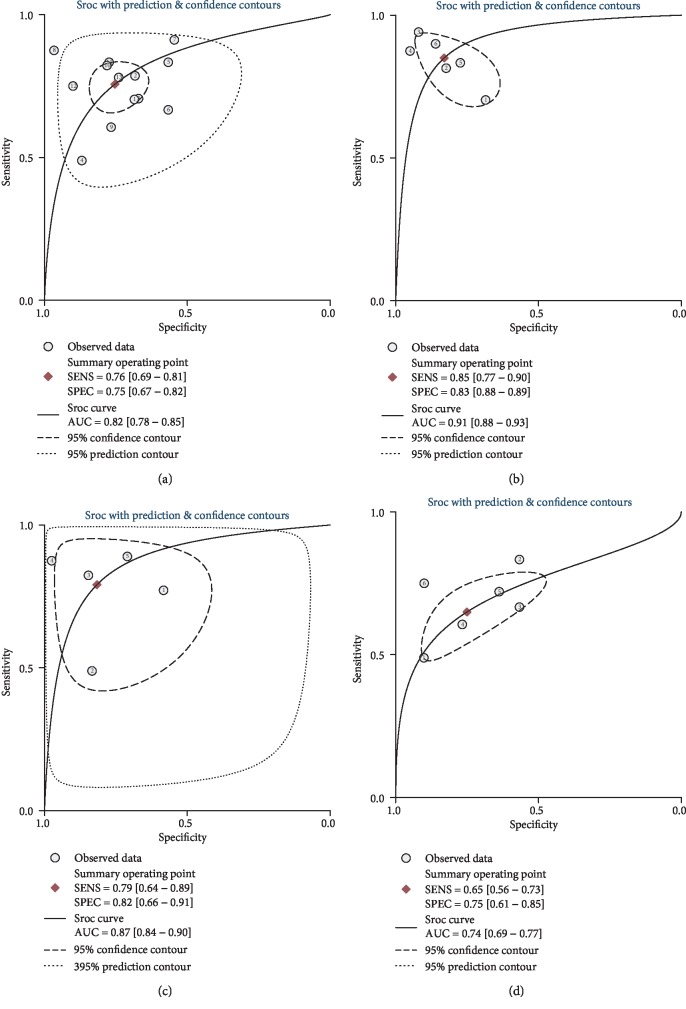
ROC curves for the diagnostic performance of circulating miR-122 for HCC: (a) summarized ROC curves for the ability of serum miR-122 to distinguish HCC patients from overall controls; (b) summarized ROC curves for the ability of serum miR-122 to distinguish HCC patients from healthy controls; (c) summarized ROC curves for the ability of serum miR-122 to distinguish HCC patients from patients with HBV or HCV infection; (d) summarized ROC curves for the ability of serum miR-122 to distinguish HCC patients from patients with liver cirrhosis or dysplastic nodule formation.

Overall, 13 studies evaluated the diagnostic performance of serum miR-122 for HCC compared with the overall control group. The summary sensitivity was 0.76 (95% CI: 0.69–0.81), and the summary specificity was 0.75 (95% CI: 0.67–0.82; Figure 2(a)) for the ability of serum miR-122 to discriminate HCC from overall controls. Significant heterogeneity was observed for the summary sensitivity and specificity (*I*^2^ = 82.8% and 83.9%, respectively). The summary AUC was 0.82 (95% CI: 0.67–0.75), according to the synthesized ROC curve (Figure 3(a)). Subsequently, we evaluated whether the diagnostic performance of serum miR-122 differed for discrimination of HCC from the different control conditions. Pooled results from six studies [11, 12, 18, 19, 21, 23] that evaluated the diagnostic performance of serum miR-122 for HCC versus the healthy control condition showed a summary sensitivity of 0.85 (95% CI: 0.77-0.90) and specificity of 0.83 (95% CI: 0.74–0.89; Figure 2(b)). The summary AUC was 0.91 (95% CI: 0.88–0.93), according to the synthesized ROC curve (Figure 3(b)). Pooled results of five studies [11, 14, 18, 19, 22] that evaluated the diagnostic performance of serum miR-122 for HCC versus HBV or HCV infection showed a summary sensitivity of 0.79 (95% CI: 0.64–0.89) and specificity of 0.82 (95% CI: 0.66–0.91; Figure 2(c)). The summary AUC was 0.87 (95% CI: 0.84–0.90), according to the synthesized ROC curve (Figure 3(c)). Pooled results of six studies [14, 16, 17, 20, 22, 24] that evaluated the diagnostic performance of serum miR-122 for HCC versus liver cirrhosis or dysplastic nodule formation showed a summary sensitivity of 0.65 (95% CI: 0.56–0.73) and specificity of 0.75 (95% CI: 0.61–0.85; Figure 2(d)). The summary AUC was 0.74 (95% CI: 0.69–0.77), according to the synthesized ROC curve (Figure 3(d)).

### 3.4. Publication Bias

**Figure 4:**
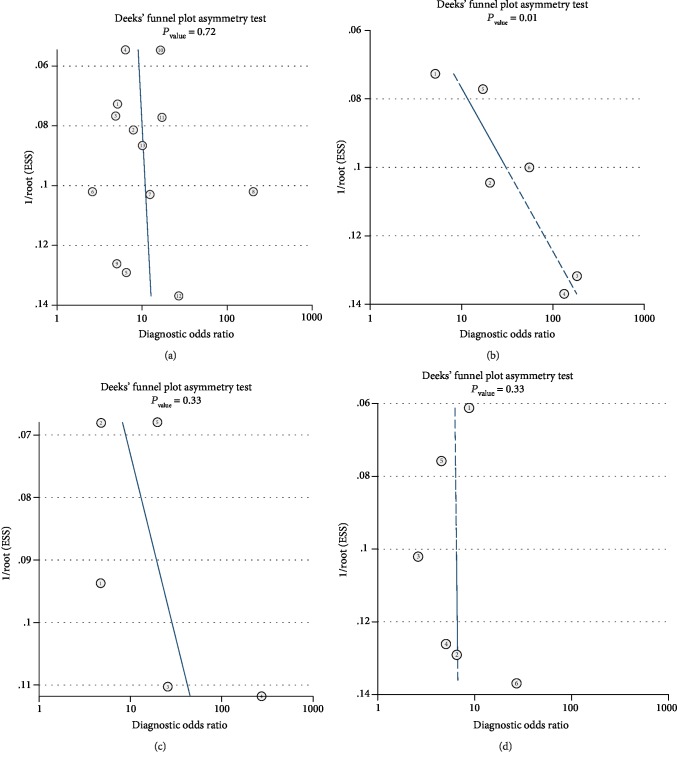
Deeks' funnel plots for the estimation of publication bias among studies evaluating the diagnostic performance of circulating miR-122 for HCC: (a) Deeks' funnel plots for meta-analysis of serum miR-122 for discriminating HCC patients from overall controls; (b) Deeks' funnel plots for meta-analysis of serum miR-122 for discriminating HCC patients from healthy controls; (c) Deeks' funnel plots for meta-analysis of serum miR-122 for discriminating HCC patients from patients with HBV or HCV infection; (d) Deeks' funnel plots for meta-analysis of serum miR-122 for discriminating HCC patients from patients with liver cirrhosis or dysplastic nodule formation.

According to Deeks' funnel plot asymmetry test, no significant publication bias was detected for this meta-analysis of the diagnostic performance of circulating miR-122 for distinguishing HCC patients from the overall control population, healthy control individuals, patients with HBV or HCV infection, and patients with liver cirrhosis or dysplastic nodule formation (*P* = 0.72, 0.17, 0.33, and 0.93, respectively, Figures 4(a)–4(d)).

## 4. Discussion

In this meta-analysis of 13 studies reporting data for 920 HCC patients and 1217 control individuals, we found that serum miR-122 conferred moderate diagnostic accuracy for HCC overall, with a summary AUC of 0.82. Subsequent analyses indicated that the serum miR-122 level confers acceptable efficacy for discriminating HCC patients from healthy controls (AUC: 0.91) or patients with HBV or HCV infection (AUC: 0.87), but less optimal efficacy for discriminating HCC patients from those with liver cirrhosis or dysplastic nodule formation (AUC: 0.74). These results suggest that measurement of serum miR-122 may be of significance for HCC surveillance in apparently healthy people or carriers of HBV or HCV, while for those with liver cirrhosis, imaging exanimations are essential.

Previous findings from experimental studies indicated that miR-122 regulates various physiological and pathological processes within hepatic cells, such as lipid metabolism, the response to drug or alcoholic hepatic injury, the response to viral infection, and hepatic fibrosis formation [10]. Accumulating evidence indicates that miR-122 expression is downregulated in the HCC tissue compared with the normal hepatic cells [29] and thus may predict poor prognosis in these patients [30]. Further studies have confirmed that miR-122 may function as a tumor suppressor during the process of hepatocarcinogenesis [31–33]. Interestingly, a higher level of circulating miR-122 has been observed in HCC patients versus those without HCC, suggesting serum miR-122 as a potential biomarker of HCC [8]. The results of our present meta-analysis further demonstrated that measurement of circulating miR-122 may confer moderate diagnostic efficacy for HCC according to histopathological examination, particularly for the discrimination of HCC patients from healthy controls or patients with HBV or HCV infection. These results are of clinical significance because they support the inclusion of serum miR-122 measurement in HCC surveillance in apparently healthy people or carriers of HBV or HCV. Moreover, for patients with already confirmed liver diseases such as liver cirrhosis, measurement of serum miR-122 may not be adequate for screening for HCC, and imaging examinations such as abdominal CT or MRI are essential for these patients. In addition, measurement of serum miR-122 for HCC surveillance has a few advantages similar to other miRNA biomarkers, as their measurement is noninvasive, stable, and reproducible [34]. The optimal protocol for circulating miR-122 measurement and its cutoff value for diagnosis of HCC in different populations deserve further evaluation.

To the best of our knowledge, only one previous meta-analysis evaluated the diagnostic role of miR-122 for HCC. This meta-analysis only included four available studies published before 2015 and concluded that miR-122 may be used to distinguish HCC patients from healthy controls. Our meta-analysis included a total of 13 studies with 920 HCC patients and 1217 controls. The larger datasets enable our study to confirm the discriminating role of circulating miR-122 for HCC from healthy controls, with comprehensively summarized diagnostic parameters including sensitivities, specificities, and AUCs. Compared to healthy controls, discriminating patients with HCC from high-risk population, such as those with HBV/HCV infection or liver cirrhosis or dysplastic nodule formation, is of more clinical importance [35, 36]. Although previous meta-analysis suggested a discriminating role of circulating miR-122 for HCC from healthy controls, its ability for the discriminating of HCC from high-risk population remains undetermined. Our meta-analysis showed that miR-122 may be useful to discriminate HCC from patients with HBV or HCV infection (AUC: 0.87), but with less optimal efficacy for discriminating HCC patients from those with liver cirrhosis or dysplastic nodule formation (AUC: 0.74). These results demonstrated that serum miR-122 may be of significance for HCC surveillance in apparently healthy people, but for patients with liver cirrhosis or dysplastic nodule formation, additional strategies are required.

Our study also has a few limitations. First, the heterogeneity among the included studies was considerable based on the *I*^2^ statistics and results of Cochran's *Q* tests. Differences in study characteristics, such as comorbidities of the patients, miR-122 cutoff values, and internal controls used during quantitative PCR, may contribute to the heterogeneity. Unfortunately, we were unable to explore the influences of the above factors on the results of the meta-analysis, because these factors were rarely reported in the included studies. Secondly, the diagnostic performance of circulating miR-122 for HCC was only moderate based on our results. Strategies to improve this efficacy deserve investigation. For example, recent studies indicated that the measurement of miR-122 from circulating exosomes may provide better diagnostic efficacy for HCC than the measurement of miR-122 in serum [37, 38]. Other strategies such as using miRNA panels [39] or combining conventional diagnostic strategies with circulating miR-122 measurement [40] may also improve the diagnostic efficacy for HCC. Finally, because the incidence of HCC is high in Chinese patients, a large proportion of studies regarding HCC diagnosis and prevention have been performed in China, as reflected in our meta-analysis. The potential diagnostic efficacy of circulating miR-122 for HCC in patients from other countries deserves further evaluation.

In conclusion, the results of our study indicate that serum miR-122 confers moderate efficacy for discriminating HCC patients from healthy controls or patients with HBV or HCV infection, but not for discriminating HCC patients from patients with liver cirrhosis or dysplastic nodule formation. Measurement of serum miR-122 may be of significance for HCC surveillance in apparently healthy people.

## Figures and Tables

**Table 1:** Characteristics of the 13 included studies evaluating the diagnostic performance of miR-122 for HCC.

Author, year	Country	Number of HCC patients	Mean age (years) of HCC patients	Male (%) among HCC patients	Control population	Number of controls	Mean age (years) of controls	Male (%) among controls	Sample type	miRmeasurement method	QUADAS score
Xu, 2011 [12]	China	101	NR	77.2	HC	89	NR	76.4	Serum	RT-qPCR	9
Qi, 2011 [11]	China	70	49.0	78.6	HBV, HC	82	42.0	74.1	Serum	RT-qPCR	9
Luo, 2013 [13]	China	85	53.9	82.4	HBV, HC	85	50.8	81.2	Serum	RT-qPCR	8
Tan, 2014 [14]	China	135	53.6	83.0	LC, HBV	222	40.5	68.2	Serum	RT-qPCR	10
El-Garem, 2014 [16]	Egypt	30	60.3	83.3	LC	30	55.1	70.0	Serum	RT-qPCR	6
Hung, 2016 [17]	China	120	58.5	80.0	DN	30	60.3	80.0	Serum	RT-qPCR	11
Ali, 2017 [18]	Egypt	34	NR	76.5	HCV, HC	77	NR	71.6	Serum	RT-qPCR	8
Amr, 2017a [19]	Egypt	40	52	82.5	HCV, HC	60	50.0	83.0	Serum	RT-qPCR	9
Caviglia, 2017 [20]	Italy	33	63.0	87.9	LC	30	54.2	63.3	Serum	RT-qPCR	11
Trung, 2018 [22]	Vietnam	118	55.6	91.2	LC, HBV, HC	288	43.1	81.6	Serum	RT-qPCR	11
An, 2018 [21]	China	84	52.7	67.8	HC	84	51.5	59.7	Serum	RT-qPCR	8
Weis, 2019 [24]	Australia	20	58.3	75.0	LC	40	51.9	67.5	Serum	RT-qPCR	12
Dai, 2019 [23]	China	50	48.6	70.0	LC, HBV, HC	100	46.1	70.0	Serum	RT-qPCR	8

HCC: hepatocellular carcinoma; NR: not reported; HC: healthy controls; HBV: hepatitis B virus infection; HCV: hepatitis C virus infection; LC: liver cirrhosis; DN: dysplastic nodule; RT-qPCR: reverse transcription quantitative real-time polymerase chain reaction; QUADAS: Quality Assessment of Diagnostic Accuracy Studies.

## Data Availability

The data used to support the findings of this study are available from the corresponding author upon request.
